# Development and validation of a nomogram for predicting immunotherapy outcomes in lung cancer patients using clinical and blood biomarkers

**DOI:** 10.1186/s12885-025-14559-1

**Published:** 2025-08-22

**Authors:** Ting Ouyang, Fei Zhang, Yan Yang, Tianwei Luo, Ao Chen, Ning Han, Jun Qian, Xiaoyuan Chu, Chao Chen, Mi Yang

**Affiliations:** 1 Department of Oncology, Nanjing Drum Tower Hospital, Nanjing Drum Tower Hospital Clinical College, Nanjing University of Chinese Medicine, Nanjing, China; 2 Department of Clinical Laboratory, Jinling Hospital, Medical School of Nanjing University, Nanjing, China; 3 Department of Medical Management, Jinling Hospital, Medical School of Nanjing University, Nanjing, China; 4 Department of Oncology, Jinling Hospital, Nanjing Medical University, Nanjing, China; 5 Department of Oncology, Jinling Hospital, Affiliated Hospital of Medical School, Nanjing University, Nanjing, 210000 China; 6 Department of Oncology, Affiliated Hospital of Nanjing University of Chinese Medicine, Nanjing, China; 7 The Comprehensive Cancer Centre of Nanjing Drum Tower Hospital, The Affiliated Hospital of Nanjing University Medical School, Nanjing, 210000 China

**Keywords:** Lung cancer, Immune checkpoint inhibitors, Prognostic biomarkers, Nomogram

## Abstract

**Background:**

Lung cancer remains the leading cause of cancer-related mortality worldwide. While immune checkpoint inhibitors (ICIs) have improved survival outcomes for some patients, their efficacy and adverse effects vary significantly. Thus, developing accurate and practical prognostic tools is essential to optimize treatment decision-making.

**Methods:**

This retrospective study analyzed 436 lung cancer patients treated with ICIs, who were randomly divided into training (70%) and validation (30%) cohorts. Independent prognostic factors for overall survival (OS) and progression-free survival (PFS) were identified using LASSO regression and multivariate Cox regression. Nomograms were constructed based on clinical and blood biomarkers. Model performance was assessed using the concordance index (C-index), ROC curve, calibration curve, and decision curve analysis (DCA). Kaplan–Meier analysis validated patient stratification.

**Results:**

The key independent predictive factors for OS and PFS included neutrophil-to-lymphocyte ratio (NLR), previous surgery, liver metastasis, clinical stage, treatment lines, and treatment response evaluation. The nomograms achieved C-index values of 0.709 (OS) and 0.730 (PFS) in the training cohort, with validation C-indexes of 0.655 (OS) and 0.694 (PFS). The ROC curves demonstrated good predictive accuracy for 12-, 24-, and 36-month outcomes. High-risk patients exhibited significantly shorter median OS and PFS (*P* < 0.001).

**Conclusion:**

The nomograms developed in this study, integrating clinical and blood biomarkers, provide a cost-effective, simple, and accurate tool for predicting the prognosis of lung cancer patients receiving ICIs treatment, to facilitate personalized clinical decision-making.

## Background

Lung cancer, a major malignancy, poses a significant threat to human health and is responsible for the highest rates of illness and death worldwide [1]. In 2022, the Global Cancer Epidemiology Statistics (GLOBOCAN) estimated approximately 20 million new cases of lung cancer worldwide, resulting in about 9.7 million deaths [1]. According to the National Cancer Center, approximately 1.06 million new lung cancer cases and 733,300 deaths were reported in China, establishing it as the country's most deadly malignancy [2]. Immune checkpoint inhibitors (ICIs) targeting PD-(L)1 have emerged as a cornerstone therapy for patients lacking other targeted options [3–5]. However, response rates vary significantly, with only a subset of patients deriving long-term benefits [5–7]. Identifying reliable predictive biomarkers is essential to distinguish responders from non-responders and guide treatment decisions.

In lung cancer, molecular indicators such as tumor PD-L1 expression, tumor mutational burden (TMB), microsatellite instability (MSI), and the presence of tumor-infiltrating immune cells are utilized to assess susceptibility to PD-(L)1 inhibitors [8–10]. These markers collectively inform the immune landscape of a tumor and help predict therapeutic responses. However, these markers inadequately capture the full variability in outcomes, likely due to the complex nature of the immune response to cancer [11, 12].

The tumor microenvironment (TME) plays a pivotal role in modulating immune responses to ICIs. Components of the TME, including immune cell infiltration, cytokine profiles, and stromal composition, critically influence immunotherapy efficacy. For instance, immunosuppressive elements such as regulatory T cells (Tregs) and myeloid-derived suppressor cells (MDSCs) within the TME can attenuate anti-tumor immunity, while high CD8 + T cell infiltration correlates with improved outcomes [13–15]. Recent studies emphasize the dynamic interplay between TME heterogeneity and ICI responsiveness. For example, spatial transcriptomic analyses reveal that immune-excluded or desert TME phenotypes are associated with resistance to PD-1 inhibitors [16]. Additionally, metabolic reprogramming within the TME, such as lactate accumulation from aerobic glycolysis, may impair T cell function and reduce therapeutic efficacy [17]. These findings underscore the need to integrate TME characteristics into prognostic models to refine patient stratification.

In clinical practice, PD-L1 expression remains the most widely used biomarker for predicting the efficacy of immunotherapy. However, studies have shown only a weak correlation between PD-L1 expression levels in tumor biopsies and the overall response rate (ORR) to ICIs [18]. In lung cancer, evaluating factors such as smoking history, TMB, MSI, high expression of CTLA4, low expression of CX3CL1, and CD8 + T cell infiltration in the tumor microenvironment (TME) may provide more robust predictive capabilities for responses to anti-PD-1/PD-L1 therapies compared to histopathological PD-L1 quantification [19–21]. However, these markers have yet to be developed into a clinically robust and practical biomarker signature.

Nomograms, graphical tools integrating multiple predictors, offer a practical solution for personalized prognosis estimation [22]. This study aims to develop and validate a nomogram combining clinical and biomarker data to predict outcomes in lung cancer patients prior to ICI initiation.

## Patients and methods

### Patients

A total of 436 lung cancer patients treated with ICIs at Nanjing Jinling Hospital (January 2019–February 2024) were retrospectively analyzed. Patients were randomly allocated into training (70%) and validation (30%) cohorts.

### Inclusion and exclusion criteria

The inclusion criteria encompassed the following: (I) Patients aged ≥ 18 years with histologically confirmed lung cancer subtypes, including adenocarcinoma, squamous cell carcinoma, or small cell carcinoma; (II) Documented metastatic subtypes (e.g., liver, bone, or brain metastases) via imaging (CT/MRI) or biopsy; (III) Received ≥ 2 cycles of immune checkpoint inhibitors (ICIs). Patients with prior systemic therapies (chemotherapy, targeted therapy) were eligible if they met this criterion. Revised exclusion criteria were as follows: (I) Poor general condition (ECOG PS ≥ 3) or comorbidities (e.g., immune deficiencies) precluding tolerance to immunotherapy or chemotherapy; (II) Incomplete baseline biomarker data (NLR, PLR, CEA) or missing follow-up records; (III) Missing data > 5% (e.g., CA125 in 8% of cases), which were addressed via multiple imputation by chained equations (MICE) under the missing-at-random assumption.

### Treatment regimens

The treatment regimens for ICIs included: 293 patients (67.2%) received combined chemotherapy; 57 patients (13.1%) were treated with both chemotherapy and anti-angiogenesis therapies (AATs); 54 patients (12.4%) received only AATs; and 32 patients (7.3%) underwent immune monotherapy.

### Data collection

Baseline characteristics collected from patients included age, sex, smoking history, metastatic sites at baseline, presence of ascites, prior treatments (surgery, radiotherapy, chemotherapy, targeted therapy, and anti-angiogenic therapy), site of disease progression, histological subtypes (adenocarcinoma, squamous cell carcinoma, small cell carcinoma, and others), gene mutations, cancer staging, types of ICIs administered, immunotherapy combination regimens, treatment lines, efficacy assessments, and immunotherapy. Additional factors such as treatment adverse reaction scores, ECOG PS, neutrophil/lymphocyte ratio (NLR), platelet/lymphocyte ratio (PLR), hemoglobin levels (HGB), carcinoembryonic antigen (CEA), carbohydrate antigen 125 (CA125) and 199 (CA199), white blood cell count (WBC), time to progression, date of death.

### Evaluation of curative effect

Imaging evaluations (CT/MRI) were performed at baseline, after every two treatment cycles, and at suspected progression. Scans were independently reviewed by two radiologists blinded to clinical outcomes, with discrepancies resolved by a third reviewer. Responses were categorized per RECIST 1.1: complete response (CR), partial response (PR), stable disease (SD), or progressive disease (PD) [23]. Target lesions were measured in axial planes, and non-target lesions were assessed qualitatively. Preliminary therapy response evaluations were conducted after two treatment cycles or as medically necessary. The primary objectives of the study were to determine overall survival (OS) and progression-free survival (PFS). OS was defined as the duration from the initiation of ICIs treatment to the last follow-up or death, whereas PFS was defined as the time from the initiation of ICIs treatment until disease progression. Patient safety was evaluated, with the severity of adverse events categorized according to the National Cancer Institute's Common Terminology Criteria for Adverse Events (CTCAE) Version 5.0. Informed consent was obtained from all participants in accordance with the ethical guidelines outlined in the Declaration of Helsinki (2013 revision), ensuring that all aspects of the study respected participant autonomy and safety.

### Biomarker detection

Peripheral blood samples were collected pre-treatment. NLR and PLR used the Mindray BC-6800Plus automated hematology analyzer (Mindray Corporation, Shenzhen, China), which was calibrated, controlled, and maintained according to the manufacturer's recommendations. CEA, CA125, and CA199 levels were quantified using Maglumi X8 fully automated chemiluminescence immunoassay analyzer (New Industry Biotech, Shenzhen, China). All assays followed manufacturer protocols, with inter- and intra-assay coefficients of variation < 10%.

### Statistical analysis

Statistical analyses were conducted using R software (RRID:SCR_000432, version 4.1.3). Baseline characteristics were analyzed using Chi-square or Fisher's exact tests for categorical variables (reported as frequencies/proportions) and nonparametric tests (e.g., Mann–Whitney U) for non-normally distributed continuous variables (reported as medians with interquartile ranges). For variables with > 5% missing data (e.g., CA125 in 8% of cases), multiple imputation by chained equations (MICE) with predictive mean matching was applied, generating five imputed datasets; pooled results were derived using Rubin's rules. Prognostic factors were identified via LASSO regression, with the optimal penalty parameter (λ) determined through tenfold cross-validation. Variables with non-zero coefficients were retained, and those statistically significant (*P* < 0.05) in multivariate Cox regression were incorporated into the nomogram, presented as hazard ratios (HRs) with 95% confidence intervals (95%CIs). Multivariable models adjusted for confounders, including comorbidities (e.g., chronic obstructive pulmonary disease, diabetes), ECOG performance status, and combination therapies (chemotherapy/anti-angiogenic agents). Propensity score weighting (inverse probability of treatment weighting) was employed to balance baseline characteristics between treatment lines [24]. Model performance was evaluated using Harrell's concordance index (C-index), time-dependent ROC curves (AUC for 12-, 24-, and 36-month survival), and decision curve analysis (DCA) to assess clinical utility. Patients were stratified into low-risk and high-risk groups based on nomogram-derived thresholds. Survival differences were analyzed via Kaplan–Meier curves and log-rank tests, with statistical significance set at *P* < 0.05.

### Ethical statement

The authors are responsible for all aspects of this work in ensuring that questions related to the accuracy or integrity of any part of the work are appropriately investigated and resolved. This study adhered to the ethical principles set forth in the Declaration of Helsinki (2013 revision) and received approval from the Ethics Committee of Nanjing Jinling Hospital (No. DZQH-KYLL-23-06). All participants provided informed consent.

## Result

### Patient clinical characteristics

**Fig. 1:**
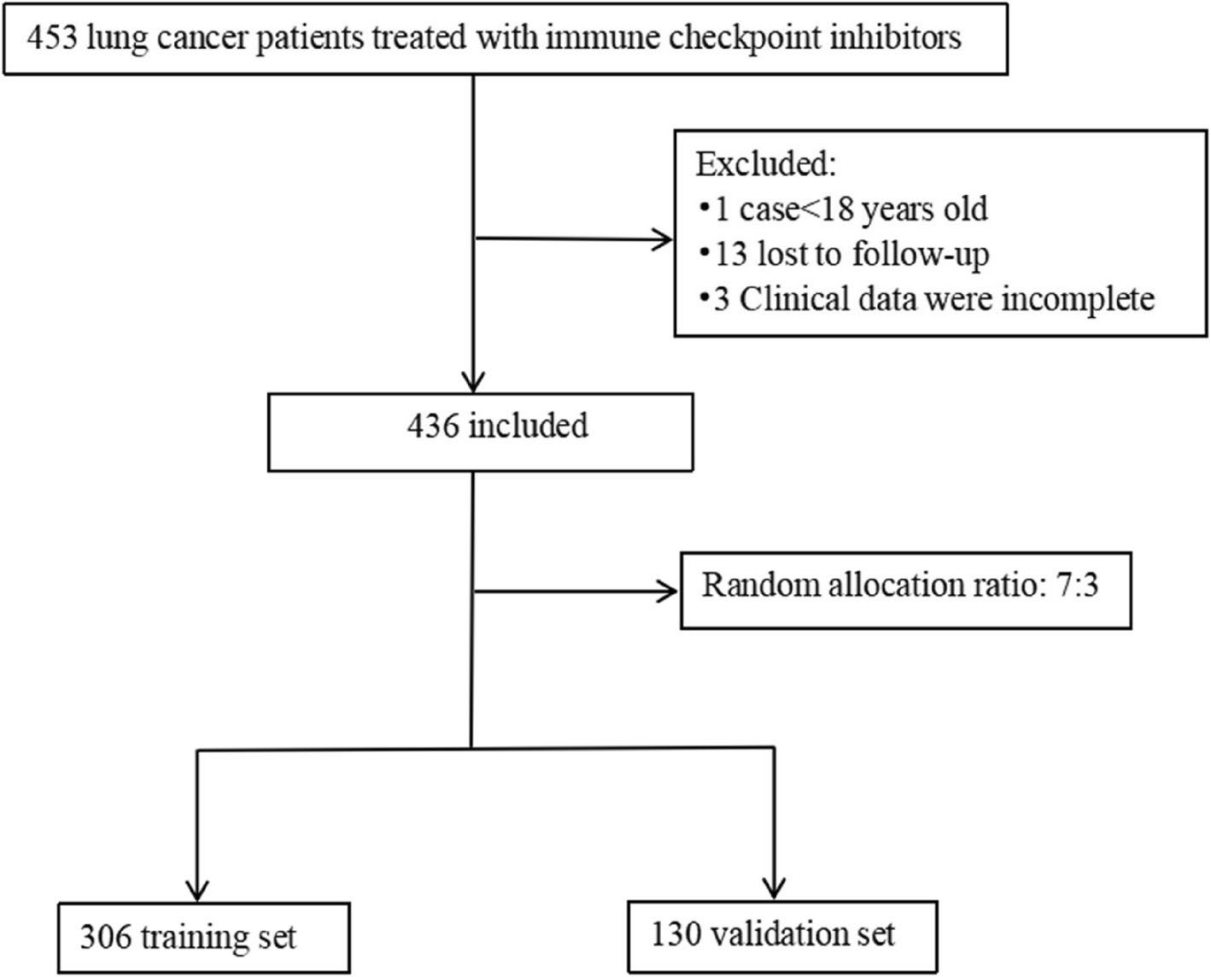
Flow chart of the study

The study included a total of 436 patients, as depicted in the flowchart presented in Fig. 1. These patients were assigned to either the training set (*n* = 306) and the validation set (*n* = 130). The age distribution revealed that 314 patients were younger than 70 years, while 122 patients were aged 70 years or older.The histological breakdown of the patient cohort showed that 209 patients (47.9%) had lung adenocarcinoma, 171 (39.2%) had squamous cell carcinoma, 38 (8.7%) had small cell lung cancer, and 18 (4.1%) had various other neoplasms. Prior treatments were as follows: 108 patients had surgery, 80 had radiation therapy, 215 had chemotherapy, 66 had targeted therapy, and 85 had antivascular therapy. Fluid accumulation prior to baseline assessment was observed in 30 patients (6.9%). PD-1 monoclonal antibody usage was: 137 patients received camrelizumab (31.4%), 23 received pembrolizumab (5.3%), 89 received sintilimab (20.4%), 144 received tislelizumab (33.0%), and 43 received other treatments (9.9%). Regarding treatment lines, 264 patients (60.6%) received first-line checkpoint inhibitors, 104 (23.9%) received second-line treatments, and 68 (15.6%) received third-line treatments. Comparative analyses indicated no significant statistical variances between the training and validation sets, confirming their comparability (*P* > 0.05).

### Selection of prognostic factors

**Fig. 2:**
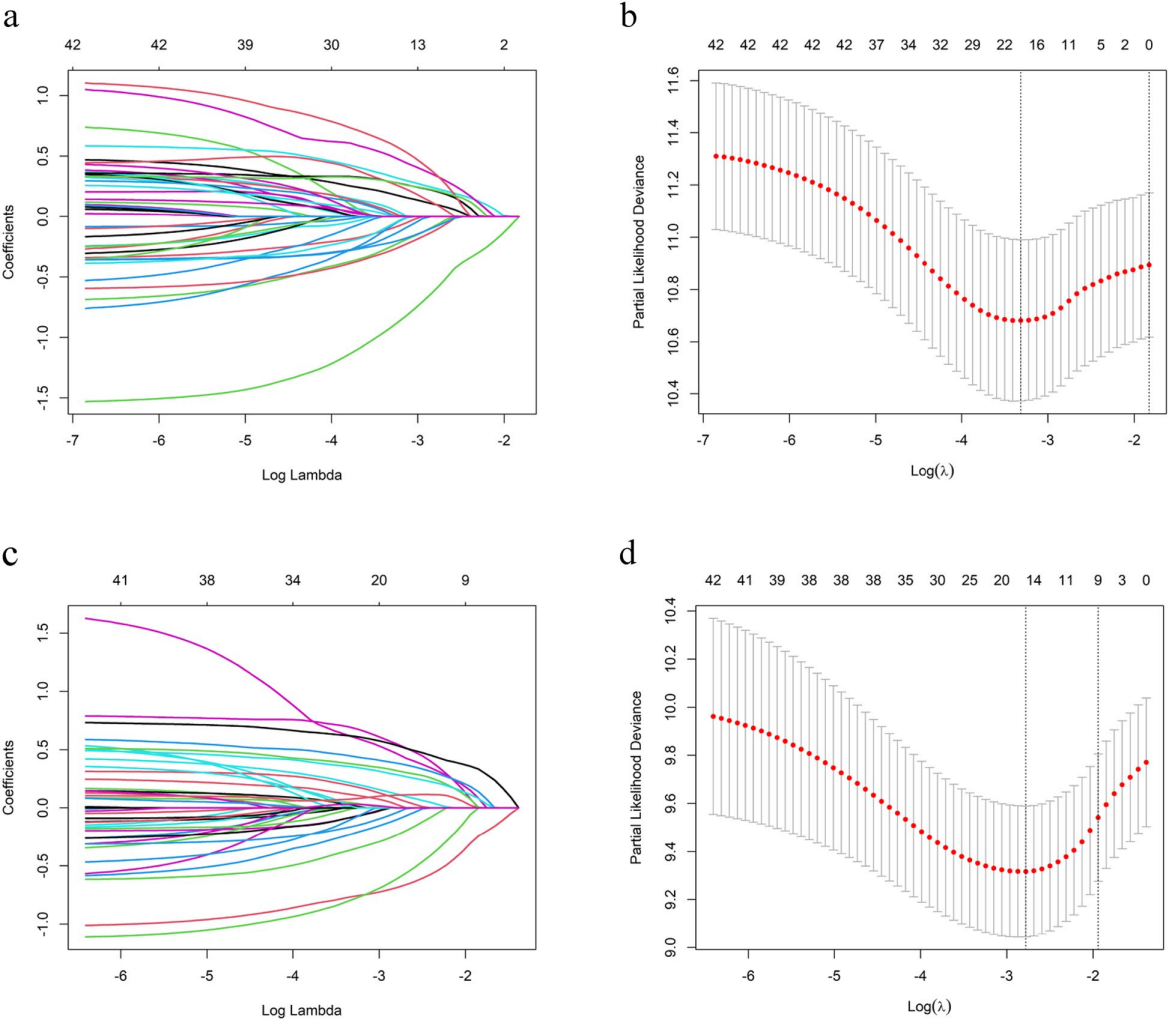
Factor selection and linear map development using clinicopathological characteristics and peripheral blood markers. **a** OS-LASSO coefficient profiles of candidate variables. **c** PFS-LASSO coefficient profiles of candidate variables. **b** and **d**, The LASSO study utilized ten-fold cross-validation to identify the ideal tuning parameter lamb λ. The non-zero coefficients established via ten-fold cross-validation are represented by the dashed and vertical lines. The initial vertical line corresponds to the smallest measured error. The second vertical line shows that the cross-validation error is within a standard error minimum

LASSO Cox regression analysis was employed to evaluate recorded clinical measures, including clinicopathological characteristics and biomarkers, with the aim of identifying significant prognostic factors, as detailed in Table 1. Significant correlations with OS were observed for smoking history, staging, surgery, radiotherapy, liver metastases, bone metastases, other metastases, ECOG PS, types of ICIs, immunotherapy regimens, treatment lines, efficacy assessment, AE grade, WBC, NLR, and CEA, as shown in Fig. 2a-b. Significant correlations with PFS were found for ascites, lung metastases, liver metastases, bone metastases, lymph node metastases, other metastases, treatment lines, and efficacy assessment, as detailed in Fig. 2c-d. Prospective prognostic factors for OS and PFS were identified by incorporating clinical indicators from LASSO Cox regression into multivariate Cox proportional hazards regression analysis. Multivariate Cox regression analysis was conducted to identify independent predictors of OS and PFS. Patients with an NLR < 5.06, prior surgery, clinical stage II-III, fewer than two lines of therapy, SD and PR efficacy ratings, mild adverse events (grade 1/2), and no liver metastases experienced improved survival benefits. Similarly, patients with fewer than two lines of therapy, SD and PR efficacy assessments, and no initial ascites, lung, bone, lymph node, or other metastases demonstrated superior survival advantages, as shown in Table 3.

**Table 1:** Baseline characteristics

Variables	Total (*n* = 436)	Training set (*n* = 306)	Validation set (*n* = 130)	*p*
Age				0.255
≤ 70	314 (72.02)	215 (70.26)	99 (76.15)	
> 70	122 (27.98)	91 (29.74)	31 (23.85)	
CEA(ug/L)				0.541
≤ 9.8	150 (34.40)	102 (33.33)	48 (36.92)	
> 9.8	286 (65.60)	204 (66.67)	82 (63.08)	
CA125(u/ml)				0.925
≤ 35	91 (20.87)	63 (20.59)	28 (21.54)	
> 35	345 (79.13)	243 (79.41)	102 (78.46)	
CA199(u/ml)				0.999
≤ 37	204 (46.79)	143 (46.73)	61 (46.92)	
> 37	232 (53.21)	163 (53.27)	69 (53.08)	
WBC((× 10^9^/L)				0.999
≥ 3.5	405 (92.89)	284 (92.81)	121 (93.08)	
< 3.5	31 (7.11)	22 (7.19)	9 (6.92)	
HGB(g/L)				0.541
≥ 115	286 (65.60)	204 (66.67)	82 (63.08)	
< 115	150 (34.40)	102 (33.33)	48 (36.92)	
NEU_LYM				0.900
≤ 5.06	292 (66.97)	206 (67.32)	86 (66.15)	
> 5.06	144 (33.03)	100 (32.68)	44 (33.85)	
PLT_LYM				0.999
≤ 205.99	241 (55.28)	169 (55.23)	72 (55.38)	
> 205.99	195 (44.72)	137 (44.77)	58 (44.62)	
Types of ICIs				0.378
Carrilizumab	137 (31.42)	96 (31.37)	41 (31.54)	
Pembrolizumab	23 (5.28)	15 (4.90)	8 (6.15)	
Sindellizumab	89 (20.41)	66 (21.57)	23 (17.69)	
Tirellizumab	144 (33.03)	104 (33.99)	40 (30.77)	
Other	43 (9.86)	25 (8.17)	18 (13.85)	
Gender				0.739
Male	353 (80.96)	246 (80.39)	107 (82.31)	
Female	83 (19.04)	60 (19.61)	23 (17.69)	
Smoking history				0.663
No	206 (47.25)	142 (46.41)	64 (49.23)	
Yes	230 (52.75)	164 (53.59)	66 (50.77)	
Surgery				0.753
No	328 (75.23)	232 (75.82)	96 (73.85)	
Yes	108 (24.77)	74 (24.18)	34 (26.15)	
Radiotherapy(baseline)				0.999
No	356 (81.65)	250 (81.70)	106 (81.54)	
Yes	80 (18.35)	56 (18.30)	24 (18.46)	
Chemotherapy				0.737
No	221 (50.69)	153 (50.00)	68 (52.31)	
Yes	215 (49.31)	153 (50.00)	62 (47.69)	
Targeted therapy				0.595
No	370 (84.86)	262 (85.62)	108 (83.08)	
Yes	66 (15.14)	44 (14.38)	22 (16.92)	
Antiangiogenic therapy				0.310
No	351 (80.50)	242 (79.08)	109 (83.85)	
Yes	85 (19.50)	64 (20.92)	21 (16.15)	
Histological subtype				0.232
Adenocarcinoma	209 (47.94)	156 (50.98)	53 (40.77)	
Squamous cell carcinoma	171 (39.22)	115 (37.58)	56 (43.08)	
Small cell lung cancer	38 (8.71)	24 (7.84)	14 (10.77)	
Other	18 (4.13)	11 (3.59)	7 (5.38)	
Stage				0.866
II-III	110 (25.23)	76 (24.84)	34 (26.15)	
IV	326 (74.77)	230 (75.16)	96 (73.85)	
Genetic mutation				0.413
No	94 (21.56)	71 (23.20)	23 (17.69)	
Yes	58 (13.30)	41 (13.40)	17 (13.08)	
Unknow	284 (65.14)	194 (63.40)	90 (69.23)	
Treatment Plan				0.451
ICIs + chemotherapy	293 (67.20)	202 (66.01)	91 (70.00)	
ICIs + chemotherapy + AATs	57 (13.07)	39 (12.75)	18 (13.85)	
ICIs + AATs	54 (12.39)	43 (14.05)	11 (8.46)	
ICIs	32 (7.34)	22 (7.19)	10 (7.69)	
Lines				0.858
1	264 (60.55)	186 (60.78)	78 (60.00)	
2	104 (23.85)	71 (23.20)	33 (25.38)	
> 2	68 (15.60)	49 (16.01)	19 (14.62)	
ECOG PS				0.438
0 ~ 1	379 (86.93)	263 (85.95)	116 (89.23)	
> 2	57 (13.07)	43 (14.05)	14 (10.77)	
Efficacy assessment				0.502
PD	116 (26.61)	82 (26.80)	34 (26.15)	
SD	261 (59.86)	179 (58.50)	82 (63.08)	
PR	59 (13.53)	45 (14.71)	14 (10.77)	
AE grade				0.863
0	209 (47.94)	147 (48.04)	62 (47.69)	
1 ~ 2	182 (41.74)	126 (41.18)	56 (43.08)	
3 +	45 (10.32)	33 (10.78)	12 (9.23)	
Ascites				0.818
No	406 (93.12)	286 (93.46)	120 (92.31)	
Yes	30 (6.88)	20 (6.54)	10 (7.69)	
Lung metastases				0.784
No	344 (78.90)	243 (79.41)	101 (77.69)	
Yes	92 (21.10)	63 (20.59)	29 (22.31)	
Liver metastases				0.076
No	408 (93.58)	291 (95.10)	117 (90.00)	
Yes	28 (6.42)	15 (4.90)	13 (10.00)	
Bone metastases				0.236
No	389 (89.22)	269 (87.91)	120 (92.31)	
Yes	47 (10.78)	37 (12.09)	10 (7.69)	
Lymph node metastases				0.999
No	380 (87.16)	267 (87.25)	113 (86.92)	
Yes	56 (12.84)	39 (12.75)	17 (13.08)	
Other metastases				0.831
No	373 (85.55)	263 (85.95)	110 (84.62)	
Yes	63 (14.45)	43 (14.05)	20 (15.38)	
Radiotherapy				0.563
No	302 (69.27)	215 (70.26)	87 (66.92)	
Yes	134 (30.73)	91 (29.74)	43 (33.08)	

*WBC* White blood cell count, *CEA* Carcinoembryonic antigen, *NEU_LYM* Neutrophil/lymphocyte ratio, *PLT_LYM* Platelet/lymphocyte ratio, *HGB* Hemoglobin, *CA125* Carbohydrate antigen 125, *CA199* Carbohydrate antigen 199, *AATs* Anti-angiogenesis therapies, *ECOG PS* Eastern Cooperative Oncology Group Performance Status, *AE* Adverse event, *PD* Progressive disease, *PR* Partial response, *SD* Stable disease

### Build OS and PFS nomogram

**Fig. 3:**
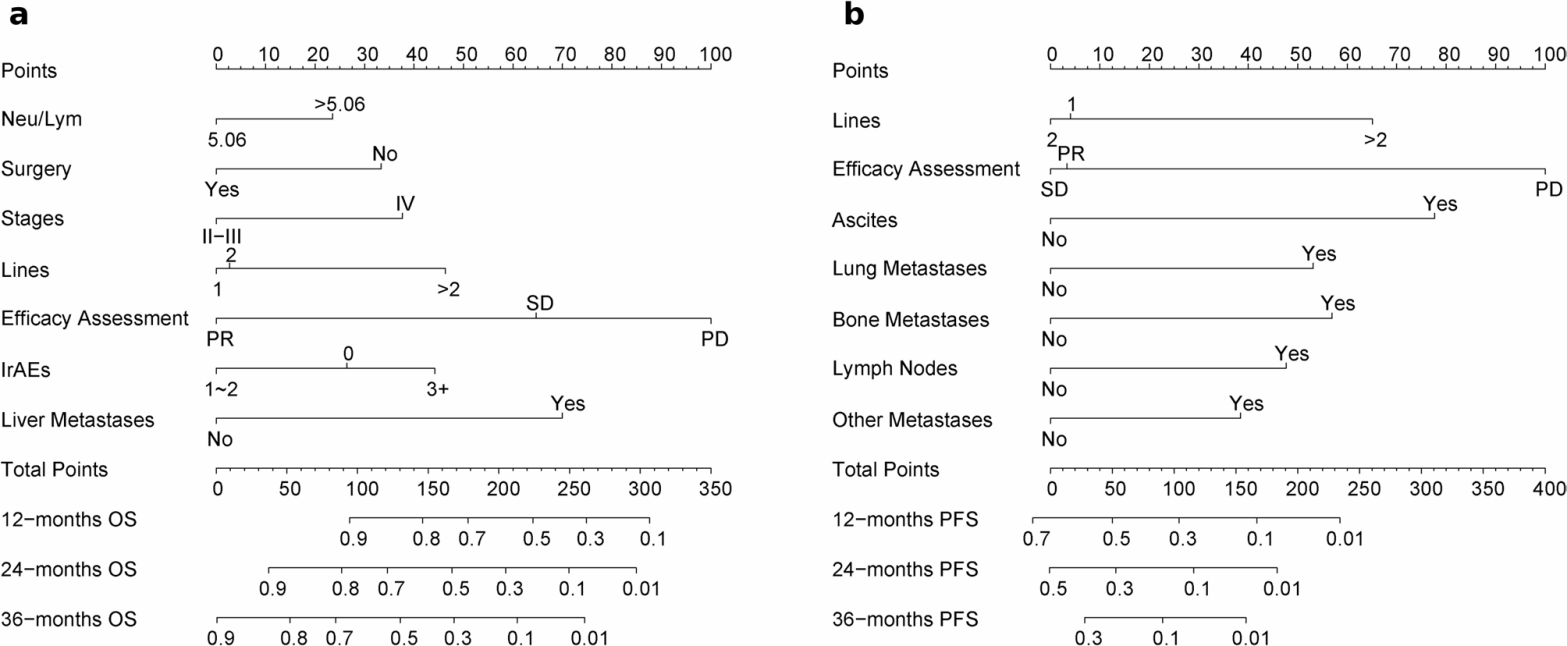
Nomograms for predicting 12-, 24-, and 36-month OS (**a**) and PFS (**b**) in lung cancer patients. NLR: Neutrophil to lymphocyte count ratio

Based on the multivariate Cox regression findings (Tables 2 and 3), OS Prediction nomogram 1 was developed using NLR, surgery, staging, treatment lines, efficacy assessment, AE grade, and liver metastases. PFS Prediction nomogram 2 was constructed with variables such as ascites, lung, bone, lymph node, other metastases, treatment lines, and efficacy assessments. Patients were randomly allocated to the training set or the validation set in a 7:3 ratio. The training set was critical for constructing the prediction model, while the validation set was used for internal validation. Figure 3 shows nomograms forecasting OS and PFS at 12, 24, and 36 months.

**Table 2:** OS multivariate cox hazards analysis

Variables	HR	95%CI	P	Wald	B	SE
CEA						
≤ 9.8	Ref					
> 9.8	0.704	0.487–1.018	0.062	3.486	−0.351	0.188
WBC						
≥ 3.5	Ref					
< 3.5	1.326	0.706–2.493	0.38	0.769	0.282	0.322
NLR						
≤ 5.06	Ref					
> 5.06	1.538	1.045–2.263	0.029	4.772	0.430	0.197
Types of ICIs						
Camrelizumab	Ref					
Pembrolizumab	1.756	0.789–3.909	0.168	1.905	0.563	0.408
Sintilimab	0.654	0.400–1.067	0.089	2.887	−0.425	0.250
Tislelizumab	1.236	0.785–1.947	0.360	0.837	0.212	0.232
Other	1.344	0.701–2.578	0.373	0.792	0.296	0.332
Smoking history						
No	Ref					
Yes	1.377	0.965–1.964	0.078	3.115	0.320	0.181
Surgery						
No	Ref					
Yes	0.493	0.312–0.780	0.003	9.131	−0.707	0.234
Radiotherapy(baseline)						
No	Ref					
Yes	1.388	0.855–2.254	0.185	1.755	0.328	0.247
Staging						
II-III	Ref					
IV	1.781	1.124–2.822	0.014	6.030	0.577	0.235
Treatment Plan						
ICIs + chemotherapy	Ref					
ICIs + chemotherapy + AATs	1.643	0.976–2.767	0.062	3.486	0.497	0.266
ICIs + AATs	0.746	0.439–1.269	0.280	1.166	−0.292	0.271
ICIs	0.477	0.215–1.060	0.069	3.300	−0.740	0.407
Lines						
1	Ref					
2	1.234	0.751–2.028	0.407	0.687	0.210	0.254
> 2	2.634	1.531–4.533	< 0.001	12.229	0.968	0.277
ECOG PS						
0 ~ 1	Ref					
> 2	1.404	0.869–2.271	0.166	1.920	0.340	0.245
Efficacy assessment						
PD	Ref					
SD	0.568	0.387–0.833	0.004	8.381	−0.566	0.195
PR	0.213	0.104–0.435	< 0.001	18.008	−1.547	0.365
AE grade						
0	Ref					
1 ~ 2	0.681	0.468–0.990	0.044	4.042	−0.384	0.191
3 +	1.204	0.673–2.153	0.532	0.391	0.186	0.297
Liver metastases						
No	Ref					
Yes	2.514	1.217–5.195	0.013	6.199	0.922	0.370
Bone metastases						
No	Ref					
Yes	1.509	0.913–2.495	0.109	2.573	0.411	0.257
Other metastases						
No	Ref					
Yes	0.673	0.403–1.122	0.129	2.309	−0.396	0.261

*WBC* White blood cell count, *CEA* Carcinoembryonic antigen, *NEU_LYM* Neutrophil/lymphocyte ratio, *AATs* Anti-angiogenesis therapies, *ECOG PS* Eastern Cooperative Oncology Group Performance Status, *AE* Adverse event, *PD* Progressive disease, *PR* Partial response, *SD* Stable disease

**Table 3:** PFS multivariate cox hazards analysis

Variables	HR	95%CI	P	Wald	B	SE
Lines						
1	Ref					
2	0.967	0.694–1.347	0.842	0.039	−0.034	0.169
> 2	1.864	1.282–2.712	0.001	10.612	0.623	0.191
Efficacy assessment						
PD	Ref					
SD	0.368	0.267–0.508	< 0.001	36.838	−0.999	0.165
PR	0.380	0.233–0.620	< 0.001	14.979	−0.968	0.250
Ascites						
No	Ref					
Yes	2.200	1.368–3.537	0.001	10.576	0.788	0.242
Lung metastases						
No	Ref					
Yes	1.737	1.262–2.390	0.001	11.484	0.552	0.163
Liver metastases						
No	Ref					
Yes	1.260	0.757–2.098	0.374	0.790	0.231	0.260
Bone metastases						
No	Ref					
Yes	1.718	1.099–2.688	0.018	5.631	0.541	0.228
Lymph node metastases						
No	Ref					
Yes	1.587	1.089–2.313	0.016	5.786	0.462	0.192
Other metastases						
No	Ref					
Yes	1.493	1.064–2.093	0.020	5.388	0.400	0.173

*PD* Progressive disease, *PR* Partial response, *SD* Stable disease

### Validation of predictive models

**Fig. 4:**
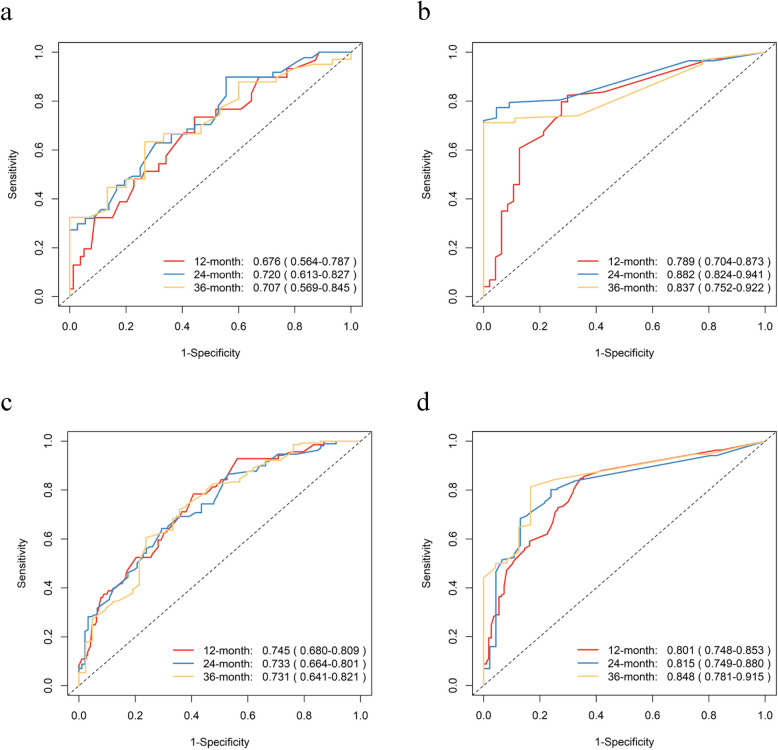
The time-dependent ROC curves of nomogram predicting OS and PFS. **a** and **b**, 12-month, 24-month, and 36-month OS and PFS in the validation set; **c** and **d**, 12-month, 24-month, and 36-month OS and PFS in the training set. ROC, receiver operating characteristic; OS, Overall Survival; PFS, Progress Free Survival

**Fig. 5:**
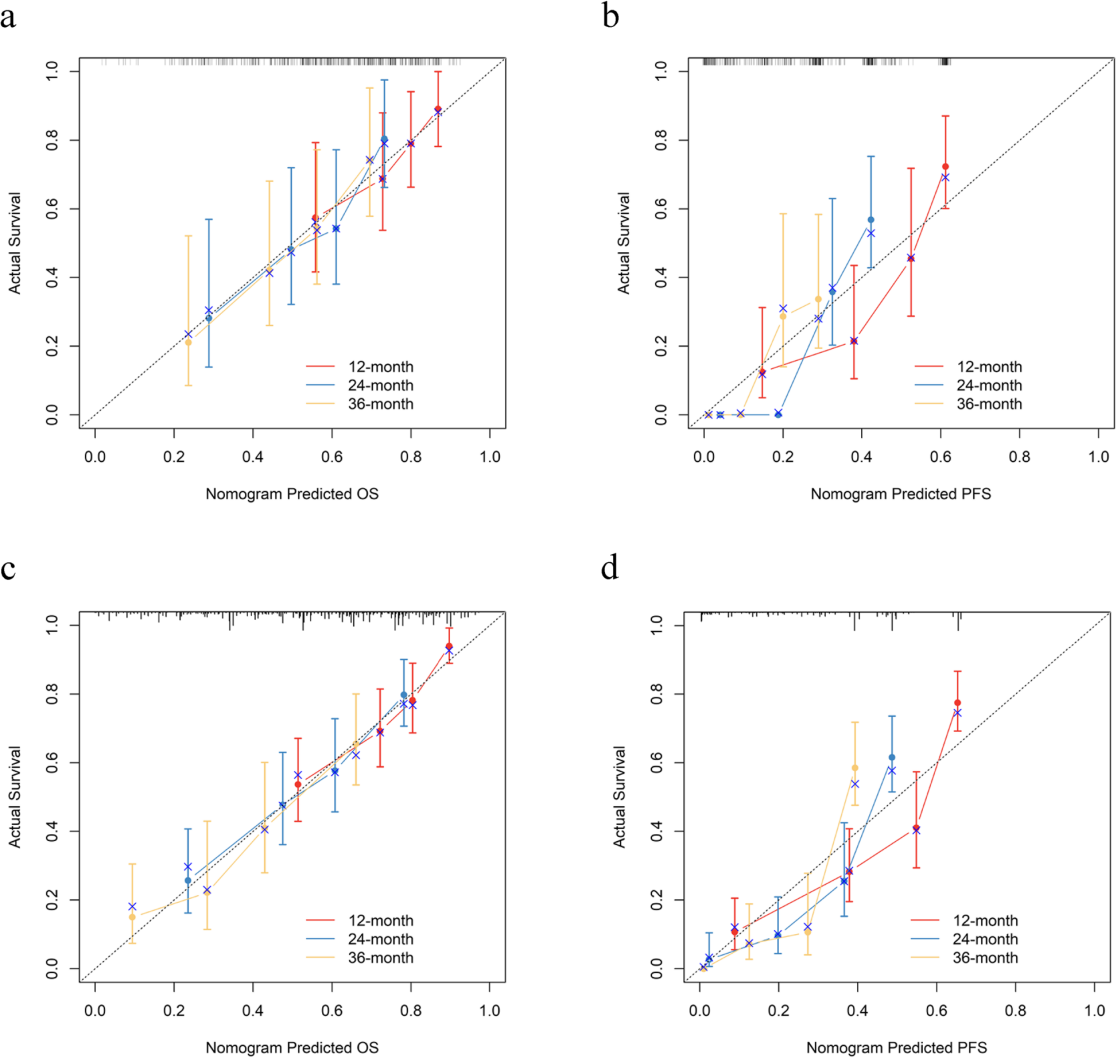
Calibration curves used to predict OS and PFS. **a** and **b**, 12-month, 24-month, and 36-month OS and PFS in the validation set; **c** and (**d**), 12-month, 24-month, and 36-month OS and PFS in the training set. OS, Overall Survival; PFS, Progress Free Survival

In the training set, the C-index values were 0.709 (95% CI: 0.666–0.752) for OS and 0.730 (95% CI: 0.698–0.763) for PFS. In the validation set, the C-index values were 0.655 (95% CI: 0.588–0.722) for OS and 0.694 (95% CI: 0.637–0.751) for PFS. The effectiveness of the risk prediction models for OS and PFS was assessed using the area under the receiver operating characteristic (ROC) curve (AUC). The ROC curves from the training set show AUC values for OS and PFS at 12, 24, and 36 months as follows: for OS, 0.745 (95% CI: 0.680–0.809), 0.733 (95% CI: 0.664–0.801), 0.731 (95% CI: 0.641–0.821); for PFS, 0.801 (95% CI: 0.748–0.853), 0.815 (95% CI: 0.749–0.880), 0.848 (95% CI: 0.781–0.915). In the validation set, AUC values for OS at 12, 24, and 36 months were 0.676 (95% CI: 0.564–0.787), 0.720 (95% CI: 0.613–0.827), 0.707 (95% CI: 0.569–0.845); for PFS, they were 0.789 (95% CI: 0.704–0.873), 0.882 (95% CI: 0.824–0.941), 0.837 (95% CI: 0.752–0.992), as shown in Fig. 4. Calibration curves from both the training and validation sets show a high level of concordance between the predicted and actual OS values, as demonstrated by nomogram 1. Similarly, Nomogram 2's predicted PFS values closely align with the observed values, as shown in Fig. 5. These results confirm the robust performance of our prediction models.

### Clinical application of nomogram

**Fig. 6:**
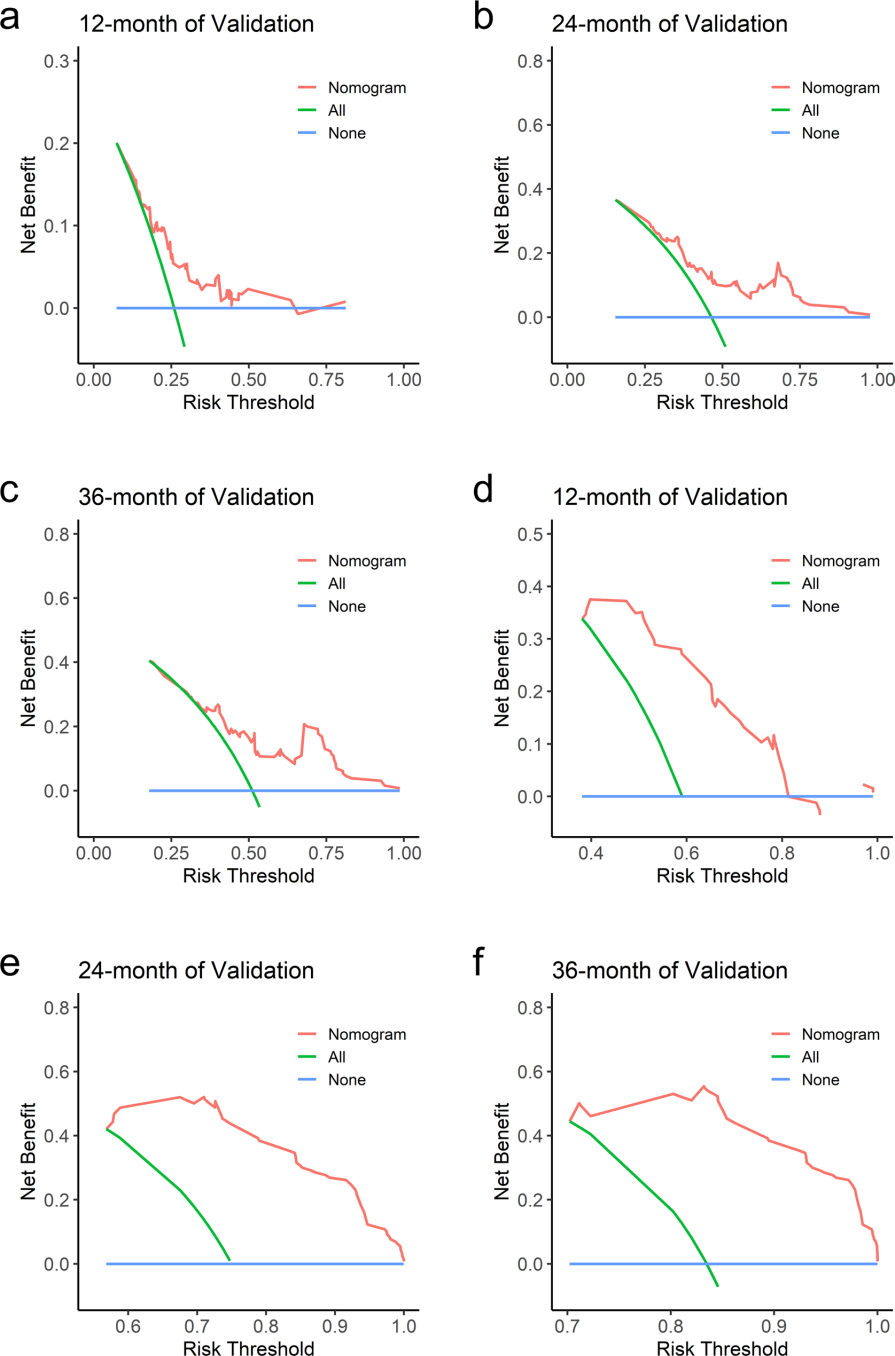
DCA of nomogram. Analysis of decision curves for 12-, 24-, and 36-month OS and PFS in the validation set. Blue line: All patients died. Green line: No patient died. Red line: nomogram model. DCA: Decision curve analysis

**Fig. 7:**
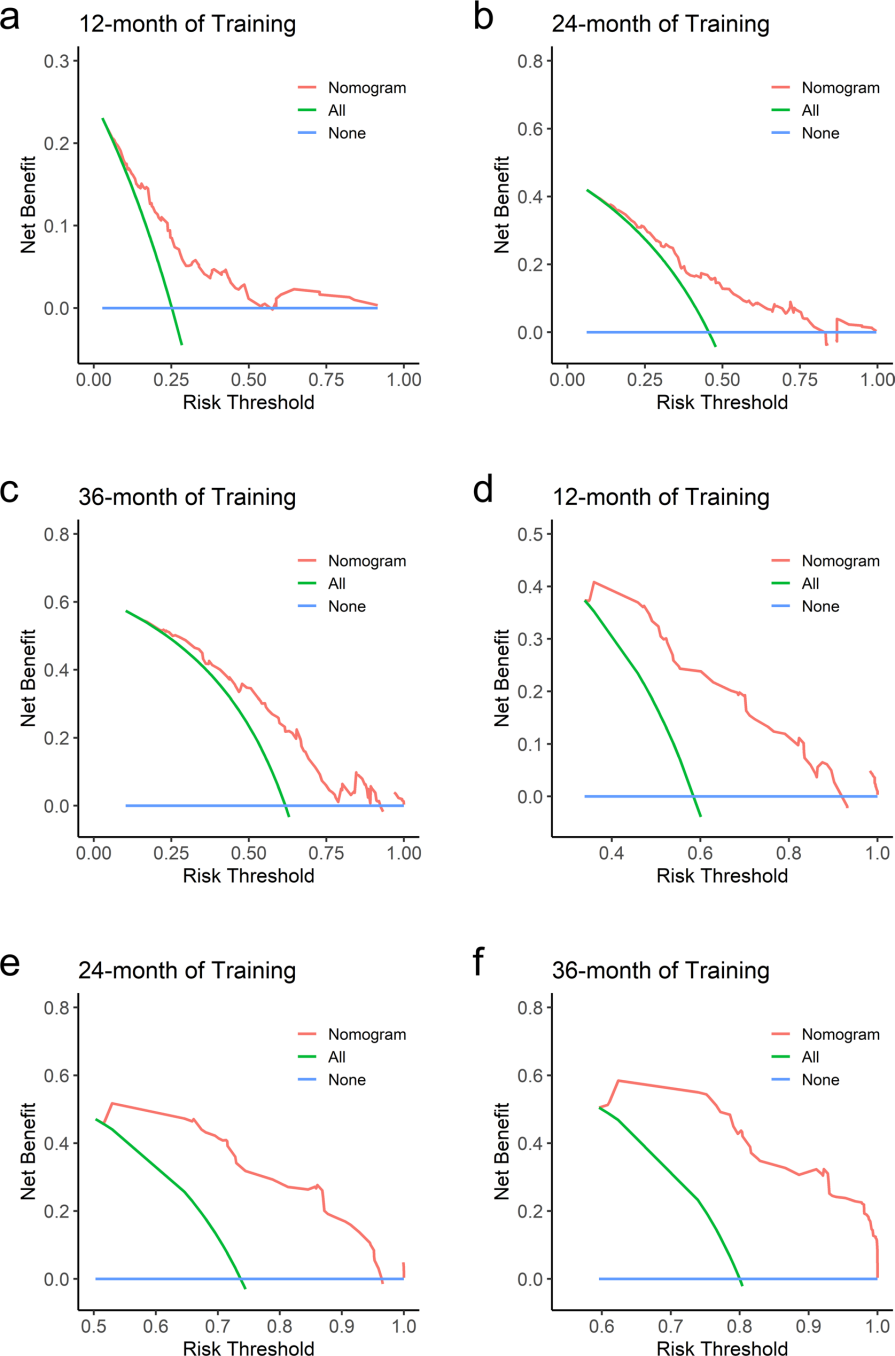
DCA of nomogram. Analysis of decision curves for 12-, 24-, and 36-month OS and PFS in the training set. Blue line: All patients died. Green line: No patient died. Red line: nomogram model. DCA: Decision curve analysis

**Fig. 8:**
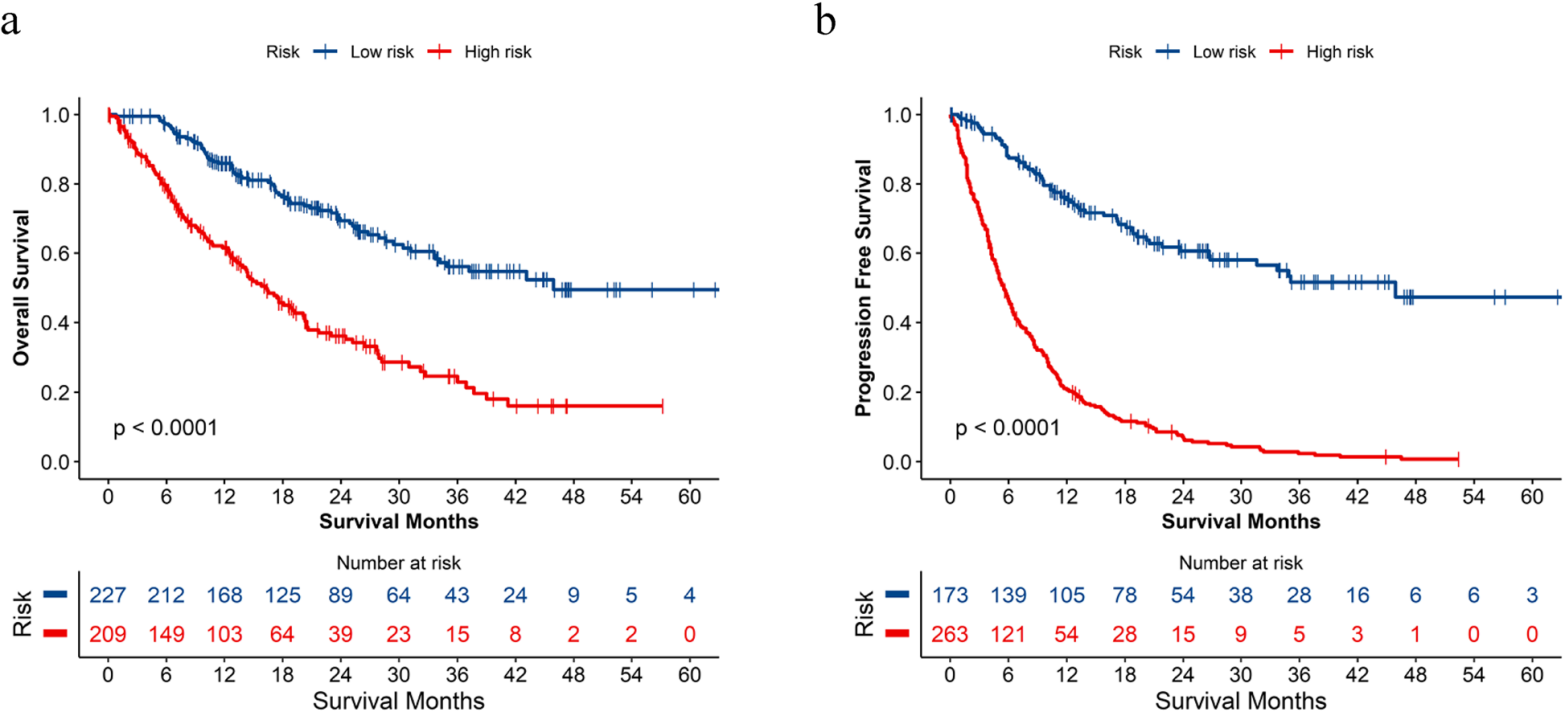
Kaplan–Meier survival analysis. Patients were divided into high and low risk groups based on risk scores and optimal cutoff values derived from nomograms for OS and PFS. **a** Kaplan–Meier curve of OS. **b** Kaplan–Meier curve of PFS

Decision Curve Analysis (DCA) was used to evaluate the clinical utility of the nomograms for OS and PFS, showing favorable outcomes in both the training and validation sets (Figs. 6 and 7). Using risk scores and cutoff values from the nomograms, patients were categorized into high-risk (OS ≥ 161.580, PFS ≥ 9.780) and low-risk (OS < 161.580, PFS < 9.780) groups. In the high-risk group, median overall survival (mOS) was 16.3 months and median progression-free survival (mPFS) was 5.5 months, with 95% confidence intervals of 0.428–0.579 and 0.438–0.560, respectively. For the low-risk group, the mOS was 45.9 months and mPFS was 35.1 months, with 95% confidence intervals of 0.400–0.611 and 0.427–0.624, respectively. The Kaplan–Meier curves validate that the risk prediction model effectively forecasts both OS and PFS (Fig. 8a and b). There was a significant correlation between the model's low-risk prediction and extended OS, as well as prolonged PFS, both with *P*-values < 0.001.

## Discuss

Accurately predicting tumor prognosis following treatment is essential for optimizing patient management and individualizing therapy. This study identified independent risk factors for OS and PFS at 12, 24, and 36 months in lung cancer patients using our training set. We developed and validated two novel nomograms to predict these outcomes at the corresponding time points. Nomogram 1 incorporates several factors, including the NLR, surgical history, clinical staging, treatment lines, efficacy assessments, immune-related adverse events (irAEs), and the presence of liver metastases. Nomogram 2 includes treatment lines, efficacy assessments, ascites, and the presence of lung, bone, lymph node, and other metastatic sites. Both nomograms demonstrated superior differentiability, achieving C-index values for OS and PFS of 0.709 and 0.730 in the training cohort, and 0.655 and 0.694 in the validation cohort. Subsequent calibration curves and decision curve analyses confirmed the predictive accuracy and clinical utility of both nomograms, with predictions closely aligning with observed outcomes.

We identified eight key predictors for lung cancer prognosis: NLR, surgical history, cancer stage, ascites, baseline metastatic sites, treatment lines, efficacy assessments, and immune-associated adverse events. The relationship between inflammation and cancer is multifaceted; NLR, as a blood-based inflammation marker, has shown effectiveness in prognostic evaluation across various cancer types [25]. Specifically, studies indicate that NLR serves as a reliable prognostic biomarker for lung cancer patients undergoing immunotherapy [26]. Our findings are consistent with a large review by Chen et al. [27], which revealed that patients with non-small cell lung cancer (NSCLC) exhibiting reduced NLR levels at weeks 6 and 12 following immunotherapy had longer PFS and OS compared to those with elevated NLR (*P* < 0.001). This emphasizes the viability of using baseline NLR as a biomarker for lung cancer prognosis, reinforcing the effectiveness of our risk prediction model. Importantly, NLR data can be readily obtained from routine blood tests, offering a cost-effective and convenient prognostic tool, although it is essential to account for potential confounding factors related to comorbidities and treatments [26, 28, 29].

Common metastatic sites for lung cancer, including bones, brain, lungs, and liver, significantly influence patient prognosis [30, 31]. Patients presenting with metastases at baseline experienced poorer outcomes. A systematic meta-analysis revealed that patients with brain, liver, or bone metastases exhibited reduced OS when treated with PD-1 inhibitors. Similarly, brain and liver metastases were associated with higher progression rates under PD-1 inhibitor treatment. Patients with multiple metastatic sites showed worse PFS than those with a single site, with no significant difference in OS observed. Multiple organ metastases are frequently correlated with poor physical condition and cancer-related cachexia. The metastatic site independently predicts survival outcomes in advanced NSCLC patients treated with PD-1 inhibitors [32]. Research consistently shows that patients with metastases at baseline experience poorer outcomes. A systematic meta-analysis corroborated that patients with brain, liver, or bone metastases exhibit reduced overall survival when treated with PD-1 inhibitors. Furthermore, multiple metastatic sites correlate with worse progression-free survival compared to a single site, though no significant differences in OS have been observed. Malignant pleural effusion was found to be an independent prognostic risk factor for PFS in our study; patients without pre-existing fluid accumulation fared better than those with it. Previous studies have identified malignant pleural effusion as a negative predictor of PFS due in part to its association with tumor-induced immunosuppression [30, 33]. Kawachi et al. proposed that vascular endothelial growth factor (VEGF) mediates pleural effusion's adverse effects by increasing vascular permeability, which can impair response to treatment. Elevated VEGF levels in patients with malignant pleural effusion have been associated with poor responses to pembrolizumab monotherapy, indicating a critical area for further investigation [30, 34, 35].

We observed that treatment lines and clinical stage were independent predictors for both PFS and OS. Notably, patients receiving immune checkpoint inhibitors (ICIs) as first-line therapy showed significant benefits. Comparative analyses revealed that pembrolizumab demonstrated a 5-year survival rate of 32% as a first-line treatment in advanced NSCLC patients with a PD-L1 expression of ≥ 50% [36]. Randomized trials have also indicated the advantage of ICIs over standard chemotherapy in second-line settings [37]. Interestingly, our findings indicated that patients treated with ICIs in the second-line setting experienced improved PFS compared to those receiving first-line treatment. This phenomenon may be attributed to the initial use of chemotherapy, which can modify the tumor microenvironment favorably, enhancing subsequent immunotherapy responses. Prior studies have shown that preconditioning with chemotherapy alters the cellular composition of the tumor microenvironment, enhancing the efficacy of immune checkpoint inhibitors [38]. Analyzing drug resistance mechanisms and optimizing immunotherapy [39] could improve outcomes in second-line treatments. Clinicians may choose a more appropriate immunotherapy or combination regimen for second-line treatment, based on patient response to first-line therapy and resistance mechanisms. Individualized therapeutic optimization can enhance the efficacy and PFS of second-line immunotherapies.

With the increasing availability and efficacy of ICI therapies, concerns regarding treatment-specific toxicities, particularly immune-related adverse events, are growing [40]. These events are often interpreted as indicators of enhanced immune responses and improved anti-tumor efficacy [41]. Consequently, numerous studies have explored whether these events could serve as indicators of ICI efficacy in cancer treatment. Emerging data suggest that irAEs may correlate with increased efficacy of ICIs in advanced malignancies such as NSCLC, indicating that patients who discontinue therapy due to irAEs might still experience therapeutic benefits [42–44]. Interestingly, our study found that patients experiencing grade 1–2 irAEs had longer OS compared to those without such events or those with severe irAEs. This observation aligns with findings from large clinical trials indicating that lower-grade irAEs are associated with better treatment outcomes, suggesting that irAEs may reflect effective immune activation and significant treatment responses [45–48].

The nomograms provide a practical tool for stratifying ICI-treated patients into low- and high-risk groups, guiding decisions on therapy escalation (e.g., combination regimens) or palliative care. For example, high-risk patients (OS < 16 months) may benefit from early integration of anti-angiogenic agents or clinical trials targeting TME immunosuppression. However, challenges include biomarker accessibility in resource-limited settings and the need for real-time NLR/CEA monitoring. While the nomogram's calculations are straightforward (summing weighted scores), integration into electronic health records requires user-friendly interfaces to minimize computational burden.

As a real-world study, our findings are subject to inherent limitations. Conducted retrospectively at a single institution, the results may have limited generalizability. The small sample size and the non-randomized nature of participant selection may introduce biases. To validate these findings, large-scale, multicenter clinical trials involving diverse patient populations are warranted. Additionally, the relatively short follow-up period necessitates ongoing monitoring and extended evaluations. Our study's approach, which assessed lung cancer patients prior to ICI treatment while considering clinical, pathological, and laboratory characteristics, generates valuable data that could assist in predicting lung cancer prognosis after immunotherapy.

## Conclusion

In conclusion, this study successfully identifies and validates critical prognostic factors for lung cancer treatment outcomes, culminating in the development of two effective nomograms. These tools hold significant promise for enhancing individualized treatment strategies and improving patient prognostication in clinical settings. Further research and validation in larger cohorts are required to ensure their broader applicability and optimization in the management of lung cancer.

## Data Availability

The data that support the findings of this study are not openly available due to reasons of sensitivity and are available from the corresponding author upon reasonable request.

## Abbreviations

PFS: Progression-free survival
OS: Overall survival
C-index: Concordance index
ROC: Receiver operating characteristic
DCA: Decision curve analyses
ECOG PS: Eastern Cooperative Oncology Group Performance Status
WBC: White blood cell count
CEA: Carcinoembryonic antigen
NLR: Neutrophil-to-lymphocyte ratio
PLR: Platelet-to-lymphocyte ratio
HGB: Hemoglobin
CA125: Carbohydrate antigen 125
CA199: Carbohydrate antigen 199
AATs: Anti-angiogenesis therapies
ICIs: Immune checkpoint inhibitors
PD-1: Anti-programmed death-1
PD-L1: Anti-programmed death ligand-1
CTLA4: Anti-cytotoxic T lymphocyte antigen 4
PD: Progressive disease
PR: Partial response
SD: Stable disease
